# TIME UNTIL THE START OF ANTIBIOTIC PROPHYLAXIS AND THE RISK OF OPEN FRACTURE INFECTION: A SYSTEMATIC REVIEW

**DOI:** 10.1590/1413-785220243202e263176

**Published:** 2024-06-24

**Authors:** João Guilherme Tavares Marchiori, Ana Paula Ferreira Nunes

**Affiliations:** 1 Universidade Federal do Espírito Santo (UFES), Department of Employee Health Care, Vitória, ES, Brazil.; 2 Universidade Federal do Espírito Santo (UFES), Department of Pathology, Postgraduate Program in Infectious Diseases, Health Sciences Center, Vitória, ES, Brazil.

**Keywords:** Antibacterial Agents, Fractures, Bone, Infection Control, Agentes Antibacterianos, Fraturas Ósseas, Controle de Infecções

## Abstract

Open fractures are highly incident injuries closely related to the modern life, in which accidents caused by motor vehicles or other machines impart high energy to bone tissue. Individual morbidity is represented by the functional impairment resultant of infection, nonunion, or vicious healing. In terms of public health, there are huge costs involved with the treatment of these fractures, particularly with their complications. One of the critical issues in managing open fractures is the use of antibiotics (ATB), including decisions about which specific agents to administer, duration of use, and ideal timing of the first prophylactic dose. Although recent guidelines have recommended starting antibiotic prophylaxis as soon as possible, such a recommendation appears to stem from insufficient evidence. In light of this, we conducted a systematic review, including studies that addressed the impact of the time to first antibiotic and the risk of infectious outcomes. Fourteen studies were selected, of which only four found that the early initiation of treatment with antibiotics is able to prevent infection. All studies had important risks of bias. The results indicate that this question remains open, and further prospective and methodologically sound studies are necessary in order to guide practices and health policies related to this matter. ***Level of Evidence II; Therapeutic Studies Investigating the Results Level of Treatment.***

## RESUMO

As fraturas expostas são lesões altamente incidentes, intimamente relacionadas à vida moderna, na qual os acidentes causados por veículos automotores ou outros aparatos transmitem alta energia ao tecido ósseo. A morbidade individual é representada pelo comprometimento funcional resultante de infecção, não-união ou cicatrização viciosa. Há enormes custos envolvidos no tratamento dessas fraturas em termos de saúde pública, principalmente quanto as complicações. Uma das questões críticas no tratamento de fraturas expostas é o uso de antibióticos, incluindo as decisões sobre quais agentes específicos devem ser administrados, a duração e o momento ideal para a primeira dose profilática. Embora as diretrizes recentes tenham recomendado o início da profilaxia antibiótica o mais rápido possível, essa recomendação parece se basear em evidências insuficientes. Em vista disso, realizamos uma revisão sistemática, incluindo estudos que abordaram o impacto do tempo até o primeiro antibiótico e o risco de resultados infecciosos. Foram selecionados 14 estudos, dos quais apenas quatro concluíram que o início precoce do tratamento com antibióticos é capaz de prevenir infecções. Todos os estudos tinham riscos importantes de viés. Os resultados indicam que essa questão permanece em aberto, sendo necessários mais estudos prospectivos e metodologicamente sólidos para orientar as práticas e políticas de saúde relacionadas a esse assunto. ***Nível de Evidência II; Estudos Terapêuticos que Investigam o Nível de Resultados do Tratamento.***

## INTRODUCTION

An open fracture is defined as a traumatic injury leading to exposure of a broken bone to external environment, with consequent contamination by microorganisms. There is always an associated soft tissue injury, the severity of which is directly related to the risks of complications, such as lack of consolidation and infection^1^ The ever-increasing incidence of open fractures reflects developments in technology in the industry, military and transport fields. Only in the US, it is estimated that up to 180.000 open fractures occur every year.^2^ Industrial accidents, gunshot wounds and, mostly, motor vehicle accidents represent the main causes of open fractures, whose incidence approaches 30 cases per 100.000 persons per year. ^3-5^ Open fractures inevitably lead to bacterial contamination of deep compartments, including subfascial soft tissues and bone. The subsequent risk of proliferation and infection is dependent on the interaction of variables such as the inoculum, host vulnerability and the lesion seriousness itself.^6^

Current paradigms in management of open fractures have included completion of bony e soft tissue reconstruction in the first 48-72 hours. Inoculum size limitation has been achieved with modernization of initial fracture management, including lavage, debridement, fixation and antibiotic prophylaxis. The infectious complication worsens the prognosis, reduces probabilities that the fracture will consolidate, increases the risk of sequelae and dysfunction, including amputation and death. In the social realm, open fractures entail exorbitant costs with hospitalizations, surgical procedures, medication, physical therapy and rehabilitation, in addition to insurance and social security costs.^7-9^

In this context, it is of great relevance to improve methods or strategies that provide a reduction in the incidence of infections associated with open fractures. Particular attention has been paid to the study of the relationship between early antibiotic (ATB) prophylaxis and the risk of infection. However, evidence is conflicting in this topic, mainly due to poor methodological quality of most studies published by now. This systematic review seeks to synthesize the body of evidence regarding this topic, in order to support relevant clinical decisions that may inform protocols and health policies addressing open fractures management.

## METHODS

### Search strategy and information sources

We initially defined the review scope using PICO acronym^10,11^ (Patient, Intervention, Comparison, Outcome), as follows: P: open fractures of any location and severity; I: early ATB after trauma; C: late ATB after trauma and O: superficial or deep infection.

Search process followed PRISMA guidelines^12^ (Preferred Reporting Items for Systematic Reviews and Meta-Analyses). An orthopedic surgeon and a microbiologist (JM and AN) independently searched the following databases: Cochrane, Embase, Pubmed, Google Scholar. Sources of gray literature were also searched, including ClinicalTrials.gov, WHO's International Clinical Trials Registry Platform (ICTRP), Networked Digital Library of Theses and Dissertations (NDLTD) and Dissertations and Theses Global. Disagreements were discussed and jointly solved. Search extended from June 2021 to February 2022, including the terms *open fractures + infection + antibiotic + timing or time or early*, with no date restriction.

### Inclusion and exclusion criteria

Randomized or non-randomized clinical trials, case-control and cohort studies were eligible, since they provided quantitative information on time to first ATB and infection endpoint.

Data extracted was registered in a Microsoft Excel spreadsheet. Complementary items were antibiotic prophylaxis regimen and its duration, the time between the fracture and the first surgical debridement, what type of osteosynthesis was used, total length of hospital stay, at what point in the follow-up the outcome occurred, which bone was fractured, open fracture classification, general demographics, presence of clinical comorbidities and missing data information.

Studies without intervention or outcome data were excluded. Regarding the design, we excluded case series, ecological studies and reviews. Others exclusions applied to duplicate, preclinical or studies with no full-text available. Only studies published in English were evaluated.

### Evaluation criteria of selected studies

We used the ROBINS-I^13^ tool for risk of bias assessment, which covers 7 essential domains (confusion, selection, missing data, classification of intervention, detection and selection bias, and bias due to deviation from the intended intervention). We chose to describe the results by separating the articles that provided recommendations from those that only indicated that early antibiotics were a current practice in trauma center. Whenever possible, we choose to group fractures with similar prognosis with the aim of improving external validity of the systematic review, since, in practice, it makes more sense to reach clinical decisions about antibiotic prophylaxis based on groups of fractures whose prognosis are similar. The main objectives of the synthesis were the identification of the methodological aspects, biases and measures of effect related to the binomial antibiotic precocity and infection. Ultimately, we meticulously investigated the selected studies, aiming at providing recommendations for practice and health policies in this matter.

## RESULTS

**Figure 1:**
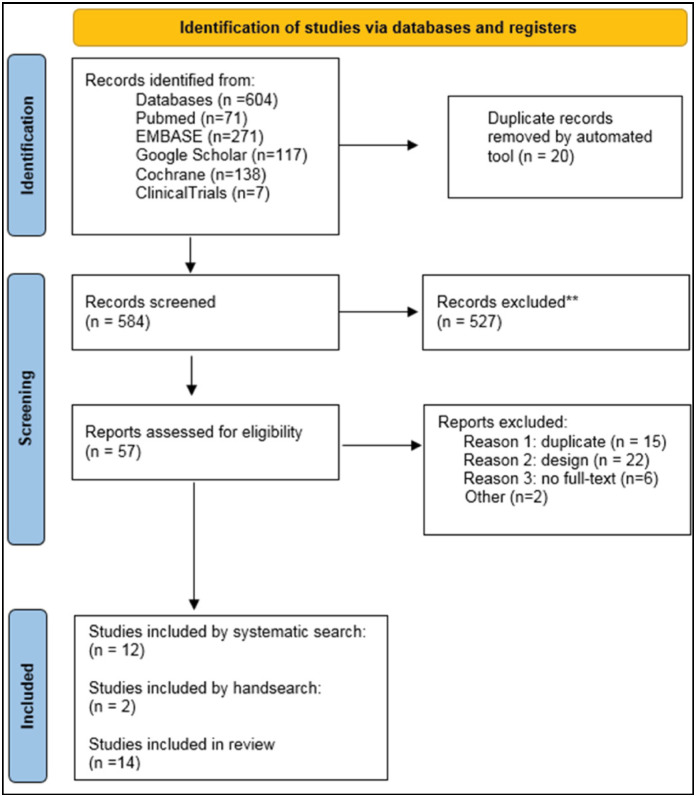
PRISMA flow diagram.

Our search initially identified 604 titles, 71 from Pubmed, 271 from Embase, 117 from Google Scholar, 138 from Cochrane and 7 from Clinicaltrials.gov. Twenty duplicate studies were automatically removed by the reference organization tool (Endnote). Of the remaining 584, 527 were excluded for not containing minimal quantitative data on the intervention or outcome. We then proceeded to a detailed analysis of 57 titles, of which 15 duplicates were additionally excluded. Others exclusions applied to 22, due to ineligible designs, 6 due to full-text unavailability and 2 for other reasons. Another 2 studies were included by handsearch. In the end, 14 studies composed the present review. (Figure 1)

Table 1. presents individual characteristics of the studies selected in the systematic review, with emphasis on results addressing the association between timing of first ATB and infectious outcome, including main author, year of publication, design, sample size, distribution of fractures by classification, risk of bias classification, information about time to first ATB and outcome.

**Table 1:** General characteristics of studies.

Study Year of publication Design	Inclusion	Sample data Timing to first ATB	Outcome definition	Risk of bias	Study risk of bias
Dellinger et al. 1988^15^ Retrospective cohort	Femur, humerus, leg bones, forearm bones All classifications Multicentric > 14y No comorbidities	N= 240 (263 fx) Minimum follow-up 21d Gt I: 25% Gt II: 47% Gt IIIA: 19% Gt IIIB: 5% Gt IIIC: 5% Method of counting time to first ATB undefined	Clinical criteria	A=M B=L C=S D=L E=S F=S G=M	S
Patzakis et al. 1989^16^ Prospective cohort	Any age Any bone	N= 1.104 or 1.390? Undefined follow-up Method of counting time to first ATB undefined	Clinical criteria, confirmed by microbiology	A=S B=S C=NI D=L E=C F=M G=M	C
Al-Arabi et al. 2008^17^ Prospective cohort	Femur, humerus, leg bones, forearm bones All classifications	N=133 Undefined follow-up Method of counting time to first ATB undefined	Clinical criteria (edema, erythema, discharge, pain), cultures when possible	A=C B=C C=S D=L E=C F=S G=M	C
Enninghorst et. al 2011^18^ Prospective cohort	> 18y, trauma center, all classifications of open diaphyseal tíbia fx	N=89 Gt I: 25% Gt II: 30% Gt IIIA: 20% Gt IIIB: 24% Gt IIIC: 1% Follow-up: 12m	Surgical debridement indication or long term systemic ATB	A=M B=L C=S D=L E=NI F=S G=M	S
Thomas et al. 2013^19^ Prospective cohort	Any age, extremity open fractures	N= 138 Follow-up: 6m 60 patients: ATB pre-hospital (helicopter) 78 patients: ATB hospital Method of counting time to first ATB: time of admission and time of trauma	Composite Endpoint (superficial or deep infection or nonunion)	A=C B=L C=S D=L E=C F=C G=M	C
Leonidou et al. 2014^20^ Prospective cohort	Open long bones fractures	N= 212 (220 fx) Analysis for first ATB included 139 patients Follow-up: until bone healing or a procedure for nonunion or infection Gt I: 36,6% Gt II: 19,9% Gt IIIA: 24,8% Gt IIIB: 18,6% Method of counting time to first ATB: time of admission and time of trauma	Purulent discharge from deep fascia, dehiscence; "radiological evidence" or cultures	A=C B=L C=S D=L E= C F=S G=M	C
Weber et al. 2014^21^ Prospective cohort	Long bones open fx of adults. All classifications	N=686 (737 fx) Gt I: 29% Gt II: 37% Gt IIIA: 21% Gt IIIB: 12% Gt IIIC: 1% Follow-up: 90d or phone interview at 12m	Surgical debridement indication or long term systemic ATB	A=M B=L C=S D=L E=S F=S G=M	S
Zumsteg et al. 2014^22^ Retrospective cohort	18y Radius and/or ulna open fx	N=200 Variable follow-up (max 6m) Gt I: 22% Gt II: 24% Gt III: 55% Data from medical records	Deep infection as an indication or surgical debridement, assessed from medical records or phone calls	A=S B=C C=S D=L E=S F=C G=M	C
Lack et al. 2015^23^ Retrospective cohort	Type III open tibia fractures	N=137 Follow-up 90d Gt IIIA: 52% Gt IIIB/IIIC: 48% Method of counting time to first ATB: time of admission and time of trauma	CDC	A=M B=L C=S D=L E=L F=S G=M	S
Johnson et al 2017.^24^ Cross-sectional	> 18y Limb and axial bones All classifications Data from medical records	N=100 1 group N= 50 before early ATB protocol. 1 group N=50 after protocol institution Undefined follow-up	Surgery indication	A=S B=C C=S D=L E=S F=S G=M	C
Assunção ALF, Oliveira de ST. 2020^25^ Prospective cohort	> 18y, trauma center, data from medical records.	N=241 Gt I: 20% Gt II: 19% Gt III: 21,6% NC: 39,4% Time from admission to first ATB	NS	A=C B=C C=S D=C E=M F=S G=M	C
Hendrickson et al 2020^26^ Retrospective cohort	Type IIIB open tíbia fx	N= 156 (159 fx) Minimum follow-up 1y Median 26 m (IQR 18-39) Method of counting time to first ATB: time of trauma	Deep infection confirmed by microbiology	A=M B=L C=L D=L E=L F=M G=M	M
Roddy et al. 2020^27^ Retrospective cohort	Upper and lower limb open fx, all classifications, data from medical records	N= 230 Minimum follow-up: 30d, endpoint assessment at 90d	CDC NHSN	A=M B=L C=S D=L E=S F=S G=M	S
Zuelzer et al. 2021^28^ Retrospective cohort	> 18y, trauma center, data from medical records, rescue sheets, Gustilo I, II, IIIA	N=127 Gt I: 27,6% Gt II: 48,8% Gt IIIA: 23,6% Minimum follow-up: 6w	CDC	A=M B=L C=S D=L E=M F=S G=M	S

A: bias due to confounding. B: selection bias. C: bias in classification of intervention. D: bias due to deviations from intended interventions. E: bias due to missing data. F: bias in measurement of outcomes. G: bias in selection of the reported result. L: low risk. M: moderate risk. S: serious risk. C: critical risk NI: no information. ATB: antibiotic. NC: not classified. NS: not specified. CDC: Centers for Disease Control. OR: Odds Ratio. ROC: Receiver Operator Characteristics. NHSN: National Healthcare Safety Network. Fx: fractures. Gt: Gustilo

Table 2. contains information on the analytical methods used, results and whether the authors made recommendations on this topic. Finally, we summarize some comments on strengths and limitations of the selected studies.

**Table 2:** Main results of studies included.

Study	Analysis	Results	Comments	Earl ATB: recommendation x usual practice
Dellinger et. al. 1988^15^	Chi-square Fisher Student's t Kaplan-Meier Logistic regression	Time to 1°ATB < 3h: 16% infected; > 3h: 17% infected. p=0,9784 Mean time to 1º ATB in infected: 2,0h (+-1,1h); non-infected: 2,2h (+-1,4h)	Method of counting the time to first ATB not informed 22% lost to follow-up at 6m	No mention about recommendation or practice
Patzakis et al. 1989^16^	Chi-square	Time to first ATB <3h (364 fx): 4,7% infected. . >3h (661 fx): 7,4% infected p= 0,087 (Yates 0,114)	No information on follow-up No control for confounding variables Method of counting the time to first ATB not informed. Dichotomization of time to first ATB variable. No information on time as a continuous variable Divergence regarding composition of the cohort (1.104 ou 1.390?) No apparent distinction between superficial and deep infection	Recommends ATB as soon as possible after lesion
Al-Arabi et al. 2007^17^	Fisher Linear Regression	Time to first ATB < 6h: 5,7% infected > 6h: 22,2% infected p=0,1144	No control for confounding variables No information regarding central tendency measures for follow-up Method of counting the time to first ATB not informed A non-specified number of more severe fx (IIIB and IIIC) lost to follow-up, with no information on their basal characteristics 80% statistical power for a reduction of 10% in infection rate	No mention about recommendation or practice
Enninghorst et al. 2011^18^	Means Student's t Mann-Whitney U Chi-square Univariate, bivariate, multiple regression	Cohort mean: 1,2h (+-0,3h) Incidence of infection: 16,8% No difference in time to first ATB between infected and non-infected	Indefinition regarding classification of intervention and outcome No missing data information	No mention about recommendation or practice
Thomas et al. 2013^19^	Fisher Chi-square Kruskal-Wallis	Pre-hospital ATB group: 60 patients (13 completed follow-up) 1 outcome (infection or nonunion [7,7%]) Hospital ATB group: 78 patients. (70 completed follow-up) 9 outcomes nonunion [12,9%]) P=1,0 60,2% lost to follow-up	No control for confounding variables Inconsistencies in classification of intervention, without proper control (potentially affects internal validity) High losses to follow-up Meticulous statistical analysis and discussion about limitations	No mention about recommendation or practice
Leonidou et al. 2014^20^	Fisher	Time to 1°ATB < 3h: 14% infected; > 3h: 12,5% infected. p=1,0	No control for confounding variables No information regarding central tendency measures for follow-up 39,6% lost to follow-up Inconsistencies in classification of intervention, without proper control (potentially affects internal validity) Inconsistencies in information of sample composition and in records of losses	Usual practice: ATB in less than 3 hours from lesion
Weber et al. 2014^21^	Medians Mann-Whitney U Simple and multiple regression	6% of infection Median to 1° ATB among infected: 2h37min. Median to first ATB among non-infected: 3h5min p=0,67 Logistic regression: OR 1,0 (IC95% 0,95-1,05)	Sound methodology Method of counting the time to first ATB not informed Few losses to follow-up. Intervention not known in 15% of patients No definite conclusion on the association of early ATB and infection, as most patients received late ATB	Usual practice
Zumsteg et al. 2014^22^	Wilcoxon Fisher Chi-square Logistic Regression	32% lost to follow-up, with no information on their basal characteristics Mean time to 1° ATB: 1,6 +− 0,9h among infected; 2,6 +− 2,2 horas among noninfected ATB < 3h: 159 patients (6% infected). ATB > 3h: 41 patients (2% infected p=0,40 10 infections (5%), on average 118 days after first stabilization	Many confounders not controlled Inconsistencies in classification of intervention High losses to follow-up Upper limb open fractures have less risk of getting infected, so big samples may be needed to investigate such associations.	No mention about recommendation or practice
Lack et al. 2015^23^	Chi-square Student's t Logistic Regression	Time to 1°ATB < 66min: 7% infected; > 66min: 25% infected p=0,0063 ROC: 66min (AUC=0,63 p=0,03) Logistic regression: ATB > 66min: OR = 3,78 (CI95% 1,26-14,11 p= -0,016)	Sound methodology and analysis Gives a cut-off time to first ATB Sample calculation for a power of 80% Late ATB is a independent predictor of infection Inconsistencies in classification of intervention, without proper control (potentially affects internal validity)	Recommends ATB as soon as possible, preferably at pre-hospital level
Johnson et al. 2017^24^	Chi-square Mann Whitney U Student's t	Time to first ATB dropped from 123,1min to 35,7min (p=0,0003). Incidence of infection = 10% for both groups	Time to first ATB counted from admission time (risk of bias due to classification of intervention) Outcome defined as indication of surgery (not precise and subjective) Follow-up not defined Small sample (few outcomes, low power)	Usual practice: first ATB as soon as possible from admission
Assunção ALF, Oliveira de ST. 2020^25^	Frequencies Chi-square	Infection: ≤ 3h from admission: 15,7% > 3 h from admission: 26,1% p = 0.0350	Confounders and co-interventions not listed	Usual practice (preoperative ATB)
Hendrickson et al. 2020^26^	Medians and IQR Logistic Regression	Time to 1° ATB: median 162 min (IQR: 120-207) Time to 1° ATB x Infection (regression analysis): Continuous: p=0,431 1h: p=0,099 3h: p=0,848	Sound methodology and analysis Main confounders accounted for, including multicollinearity tests Outcome assessed with objective criteria Potential risk of beta error, as most patients took late ATB (>2h)	Usual practice: early/pre-hospital ATB
Roddy et al. 2020^27^	Chi-square Mann Whitney U ROC Cox regression	Deep infection: 6% Median to 1° ATB in infected: 83min Median to 1° ATB non-infected: 61min p=0,053 Cut-off 120min ROC (AUC 0.62, 95% CI [0.50 – 0.75], p = 0.042) OR 2,4 [CI95% 1,1-5,7] p=0,036.	Sound methodology and analysis Gives a cut-off time to first ATB CI of AUC do not show a definitive benefit of cut-off found Small sample (low power) 130 patients missed (29%) e 78 with no information on time to first ATB (17%)	Recommends ATB as soon as possible
Zuelzer et al. 2021^28^	Chi-square Fisher ANOVA Binary regression Logistic regression ROC curve	Infection: ≤ 150 from admission: 3% > 150 from admission: 20% Odds Ratio 5.6 [95% CI 1.4 to 22.2]; p = 0.01	Sound methodology, detection bias risk, risk of bias due to classification of intervention (non-standardized sources of data)	ATB as soon as possible after lesion (practice and recommendation)

ANOVA: Analysis of Variance. ATB: antibiotic. IQR: interquartile range. OR: Odds Ratio.

## DISCUSSION

Investigation of risk factors for infection in open fractures is extremely important, given the morbidity and health costs involved in treating such complications. ^6,9^At the individual level, deep infections are difficult to treat, often incurable, with tendency to become chronic and to permanently compromise the quality of life and the work performance. This is particularly relevant when considering that open fractures are especially incident in younger and economically active age groups.^3-5^ Even with the optimization of techniques, devices and treatment protocols, infection rates can still reach 27% for type III fractures, even in specialized trauma centers.^14^

In this context of high morbidity and functional impairment, a simple and inexpensive intervention able to avoid infectious complications becomes an attractive option to be tested. Still, contemporary literature does not give the intended answers, in the face of high heterogeneity and several methodological flaws of studies published by now. In our systematic review, we chose to list such limitations, or risk of bias, both in a descriptive way, as from a standardized tool, the ROBINS-I.^13^ Bias risk assessment has shown has been especially useful in the internal comparison of studies included in the review. Generally, we observed a high risk of internal validity issues in the studies. In fact, of the 14 articles included in the systematic review, 6 were considered at serious risk of bias, 7 at critical risk and only 1 at moderate risk. The main problems encountered were substantial losses to follow-up, knowledge of the intervention at the time of assessing the outcome, and subjectivity in the classification of both the intervention and the outcome.

Regarding the follow-up, the main problems found were substantial losses, lack of definition or omission of measures of central tendency.^15,17,19,20,22,27^ In view of their designs, all studies allowed knowledge of the intervention at the time of evaluating the outcome. In others, the way of measuring the outcome was not defined^25^, or it was subjective,^15,17,18,21,22,24,27^ or without distinction between superficial and deep planes,^16^ or even taken as a composite endpoint.^19^ Another potentially serious question was the inconsistency in the way time to the first ATB was accounted for. In fact, some studies started time counting from the time of trauma, others from hospital admission, and still others from both timepoints, without performing a separate analysis for each of these situations. ^19,20,23^ For example, patients whose first dose of ATB was administered after 30 minutes after admission and who became infected were mistakenly classified as early ATB takers, as the time elapsed between the trauma and hospital admission was not accounted for. So, eventual infections in this group are mistakenly associated with early ATB, when in fact should be attributed to late intervention. The net effect is a tendency to mitigate eventual contributions of early ATB in reducing the risk of infection.

Some studies classified timing to first ATB from trauma time, ^26^ while others did so from hospital admission. ^15,24,25^ The latter situation makes time registry of first ATB earlier than in fact it was. Some studies did not define the method of accounting time to first ATB.^15-18,21,22^ We found situations of lack of balance between the comparison groups, with cases in which the vast majority of the sample either took ATB too early^18^ or too late, ^21,26^ which tends to reduce statistical power and favor the null hypothesis.

Few authors performed comprehensive control of confounders,^19,21,27,28^ and most samples were not large enough to confer adequate statistical power, or, even if there was a representative sample, the number of outcomes was small, introducing a risk of false negative associations between confounders and the endpoint. Although there were substantial limitations in all studies, we found, in the most recent publications, better methodological and analytical elaboration,^19,21,26-28^ which reflects the growing interest in clarifying the real role of early antibiotic prophylaxis in the management of open fractures.

Due to great heterogeneity, low methodological robustness and absence of randomized clinical trials on this topic, it was not possible to build a meta-analytic study, which could inadvertently compromise validity of results. However, the present review was valuable in identifying methodological gaps that can be optimized in future investigations. So, we suggest that upcoming studies carry out separate (or adjusted) analyzes to patients whose exact time of trauma is known and for those whose hospital admission is the starting time point to the first ATB. The time interval to the first ATB should be, in principle, analyzed as a continuous variable, avoiding artificial categorizations. Construction of ROC curves, from the mentioned time analysis, should be encouraged, and the data related to them, including sensitivity, specificity, AUC and respective confidence intervals, must be informed. The minimum follow-up of 3 months seems reasonable, since the vast majority of infections concentrate in this period. However, measures of central tendency and dispersion related to follow-up must be recorded in all cohorts. Those individuals lost at follow-up should be analyzed for the available data, especially the time interval to the first ATB. This is because the risk of bias due to missing data will be mitigated if the losses are balanced between patients who took early ATB and those who took it later.

Regarding the classification of outcome, we suggest that validated and objective methods are used, including, whenever possible, information on subfascial origin and microbiological results. Creative ways to prevent outcome assessors from knowing about the intervention or exposure (early or late ATB) should be implemented. All these measures tend to increase the methodological homogeneity necessary for the elaboration of future meta-analyses, something not currently feasible.

Of the 14 studies included in our review, only 4 showed a positive correlation between the interval to the first ATB and the risk of infection. ^23,25,27^ However, even though the benefits of early antibiotic prophylaxis in preventing infection are still to be confirmed, there are already centers that recommend or incorporate such practices, demonstrating that it is possible to implement antibiotic prophylaxis at a pre-hospital level. ^24,29^

It is important to consider that even studies that show benefits with a small size of effect justify efforts to implement antibiotic prophylaxis as early as possible, because it is a safe, simple and cheap intervention, so that even if the number necessary to treat (NNT) is large, the cost-risk-benefit ratio will be highly favorable. Implementation of pre-hospital systemic antibiotic prophylaxis tends to be straightforward, as first-generation cephalosporins are acceptable options for all types of fractures in the Gustilo classification^23,30-32^ and do not produce considerable risks of severe allergic reactions. In fact, even in the rare cases of truly penicillin-allergic patients, the risk of cross-allergy is only 0.5%^33,34^

Of the articles included in this systematic review, even the negative ones, there is a tendency to recommend early antibiotic prophylaxis or to indicate that such a practice is routine at the trauma center, which was the case in 8 of the 14 studies. Although the evidence is inconsistent, the biological plausibility, low costs and safety of the intervention are already sufficient arguments to justify implementation of early ATB in public health policies that deal with the pre-hospital management of open fractures.^35^

## CONCLUSION

Our study synthesized the current evidence regarding the association between time to onset of antibiotic prophylaxis and the infectious outcome, reaching the conclusion that the benefits of early use of antibiotics in open fractures are yet to be confirmed, given the low methodological quality and potential risk of bias in the studies carried out so far. However, given the safety of the intervention, the ease of its implementation, its very low cost and its biological plausibility, we believe, at least at this point, that it is reasonable to keep the trend to organize services in order to institute pre-hospital administration of ATB, and that public health policies embrace this paradigm. Well-conducted prospective studies with blinding of outcome assessors and results analysts, and with adequate statistical power, can draw definitive conclusions about the potential benefits of early antibiotic prophylaxis in the management of open fractures.
